# Effect of COVID-19 vaccination on the incidence, lethality and mortality of pregnant and postpartum women

**DOI:** 10.1371/journal.pone.0327207

**Published:** 2025-07-08

**Authors:** Marcela de Andrade Pereira Silva, Fernando Castilho Pelloso, Maria Dalva de Barros Carvalho, Rosana Rosseto de Oliveira, Constanza Pujals, Stéfane Lele Rossoni, Natan Nascimento de Oliveira, Mirella Machado Ortiz Modesto, Deise Helena Pelloso Borghesan, Ana Carolina Jacinto Alarcão, Vlaudimir Dias Marques, Marcia Edilaine Lopes Consolaro, Camila Wohlenberg Camparoto, Helena Fiats Ribeiro, Sandra Marisa Pelloso

**Affiliations:** 1 Postgraduate Program in Health Sciences, State University of Maringá, Maringá, Paraná, Brazil; 2 Curitiba Health Department, Curitiba, Paraná, Brazil; 3 Postgraduate Program in Nursing, State University of Maringá, Maringá, Paraná, Brazil; 4 Aesthetics department, Union of Catholic Colleges of Mato Grosso, Várzea Grande, Mato Grosso, Brazil; 5 Psychology department, Paraná Adventist College, Ivatuba, Paraná, Brazil; 6 Regional University Hospital of Maringá, Maringá, Paraná, Brazil; 7 Postgraduate Program in Biosciences and Physiology, State University of Maringá, Maringá, Paraná, Brazil; Fundacao Oswaldo Cruz Instituto Rene Rachou, BRAZIL

## Abstract

**Objectives:**

To analyze the incidence of Severe Acute Respiratory Syndrome (SARS), lethality and mortality of Brazilian pregnant and postpartum women infected with SARS-CoV-2, before and after vaccination against Covid-19.

**Methods:**

This is an ecological study of time series, carried out with secondary data from the Brazilian Ministry of Health, from March 2020 to April 2024. Slopes and trend changes in the time series were identified by Mann-Kendall and generalized fluctuation tests, respectively. The series was modeled using Generalized Additive Models with interaction between time and start of vaccination. A Pearson correlation was used between the number of accumulated doses and the incidence, lethality and mortality rates, in addition to comparing the average rates before and after the vaccination peak of the first dose using ANOVA.

**Results:**

Significant temporal variations were observed in the incidence rates of SARS, lethality and mortality in pregnant and postpartum women with SARS-CoV-2, before and after vaccination against Covid-19 in Brazil, with a significant decreasing trend after the start of vaccination. It was observed that time alone did not show a significant effect on the reduction of lethality and mortality rates, which occurred only when there was an interaction effect between time and the start of vaccination. The accumulated doses of the vaccine correlated with the decrease in the analyzed rates, which explained 39.18% of variation in the incidence rate, 43.34% in the lethality rate, and 34.81% in the mortality rate. The monthly averages of the incidence, lethality, and mortality rates before and after vaccination reduced significantly.

**Conclusions:**

The findings of this study indicate that vaccination against Covid-19 in Brazilian pregnant and postpartum women had a positive effect on reducing the incidence of SARS by SARS-CoV-2, lethality and mortality rates in the obstetric population.

## Introduction

According to the World Health Organization (WHO), after five years since the first case of Covid-19, there have been approximately 776 million cases and more than 7 million deaths from the disease [1]. Being among the countries with the highest number of cases, and surpassed only by the United States in the number of deaths, Brazil had also a high number of cases among the obstetric population, with a high lethality rate and an exponential increase in maternal mortality countrywide [1–4].

From March 2020 to May 2021, which corresponds to the first 15 months of the pandemic, Brazil recorded 11,247 confirmed cases of Severe Acute Respiratory Syndrome (SARS) due to SARS-CoV-2 in pregnant and postpartum women, 1,031 of which evolved to death, resulting in a lethality rate of 9.2%^3^. In the same period, 3,291 maternal deaths were registered in Brazil, which corresponded to an excess of 70% in maternal deaths observed throughout the country [4].

With the main objective of reducing morbidity and mortality caused by SARS-CoV-2, the vaccination campaign against Covid-19 started in Brazil in January 2021 [5]. However, despite the national scenario at the time, and evidence of a higher risk of severe outcomes and death in pregnant and postpartum women, this population was only inserted as a priority group for vaccination in May 2021. Incidentally, data on the efficacy and safety of the vaccine during pregnancy and the puerperium were limited [6–8].

It is estimated that vaccination against Covid-19 prevented 19.8 million deaths from the disease worldwide, with a global reduction of 63% of deaths during the first year of vaccination [9]. In Brazil, the hospitalization, lethality and mortality rate due to Covid-19, as well as the number of cases admitted to the Intensive Care Unit or who required invasive ventilatory support, reduced significantly after the start of vaccination in the country [10].

Studies conducted with the general population demonstrated a positive and substantial impact on reducing the incidence of severe cases and deaths from the disease [11–13]. Pregnant women infected with SARS-CoV-2 who had a history of vaccination tend to present mild clinical symptoms and a short hospitalization time [14].

Given the importance of monitoring changes in the impact of the disease, as well as the effectiveness of the vaccination campaign against Covid-19 in pregnant and postpartum women, the present study aims to analyze the incidence of Severe Acute Respiratory Syndrome, lethality and mortality of Brazilian pregnant and postpartum women infected by SARS-CoV-2, before and after vaccination against Covid-19.

## Methods

This is an ecological study of a time series, carried out with secondary data from the public domain made available by the Department of Statistics of the Unified Health System (DATASUS) of the Brazilian Ministry of Health. Data regarding cases and deaths from Covid-19 were extracted from the Information and Epidemiological Surveillance System for Influenza (SIVEP-Gripe), the official system for recording cases and deaths due to Severe Acute Respiratory Syndrome (SRAG) in Brazil (https://opendatasus.saude.gov.br). The database was downloaded on September 29, 2024 using the R software.

All SARS-CoV-2 SARS cases in Brazilian pregnant and postpartum women, aged between 10 and 49 years, notified between March 2020 and April 2024 were included. The age range of 10–49 years old was delimited for the study population, given that in Brazil, for research purposes, a woman of childbearing age is considered to belong to this group [15]. The analyzed cases covered the 27 federative units of Brazil, with an estimated population of 212.5 million in 2024 [16], and were investigated according to the country’s macro-regions (north, northeast, midwest, south and southeast).

To calculate the incidence and mortality rates, the number of live births in the same period and location was obtained through the Live Birth Information System (SINASC) of DATASUS, which provides data on births that occurred throughout the national territory since 1990.

The incidence rate of SARS-CoV-2 in the study population was calculated from the ratio between the number of cases of SARS-CoV-2 in pregnant and postpartum women aged 10–49 years, and the number of live births in the same place and period, multiplied by 100,000. The maternal mortality rate for Covid-19 was obtained by the ratio between the number of pregnant and postpartum women aged 10–49 years with SARS-CoV-2 who died, and the number of live births in the same place and period, multiplied by 100,000.

The lethality rate was derived from the ratio between the number of pregnant and postpartum women with SARS-CoV-2 who died, by the number of pregnant and postpartum women with SARS-CoV-2 regardless of the outcome, in the same place and period, multiplied by 100. The incidence, maternal mortality and lethality were calculated monthly, during the study period, according to the country’s macro-regions.

The number of Covid-19 vaccines administered to the obstetric population were taken from the National Panel of the Ministry of Health of Brazil (Vacinômetro COVID-19), which provides processed data on vaccination against Covid-19 in the country, daily updated by data contained in the National Health Data Network (RNDS). The data used in this study were collected in September 2024, referring to the number of 1st and 2nd doses, and 1st booster doses applied to pregnant and postpartum women in the country and its macro-regions, during the period from January 2021 to April 2024.

An exploratory analysis of the vaccine doses in the study population and the behavior of the incidence, lethality and mortality rates was carried out. The trend change points (breakpoints) were found through generalized fluctuation tests and F test (Chow Test). The Mann-Kendall test was applied to identify the slopes of the incidence, lethality and mortality time series in the period before and after the start of vaccination for the group in question (January 2021).

To model the behaviors presented by the three rates, GAM (Generalized Additive Models) models with splines were used to capture the non-linearity of the series, adjusting smooth curves that adapt to the data with greater flexibility, ideal for complex time series. The interaction effect between the time elapsed and the start of vaccination (Time:Post-Vaccination) was also considered, verifying whether the time effect is different before and after the start of vaccination. The number of knots (points where the polynomial functions change within each spline) was determined so that the model maintained a reasonable similarity with the fluctuations observed in the series.

Next, the effect of the accumulated vaccine doses was also verified, this time considering the first peak of application of the 1st dose (06/2021) as the cut-off point, where the first considerable amount of doses applied occurs. The effect of the accumulated doses was verified through graphs, comparing the first monthly peak of application of the first dose with the timeline of the rates, in addition to correlation tests between the rates and the amount of accumulated doses applied to pregnant and postpartum women.

For the correlation analysis between the accumulated doses of the vaccine and the incidence, lethality and mortality rates, the Pearson correlation test was used, adopting the reference values: weak correlation r < 0.30; moderate correlation 0.30 ≦ r < 0.60; strong correlation 0.60 ≦ r < 0.99 and perfect correlation r = 1 [17].

An ANOVA test and its Tukey post-hoc were also performed to compare the average monthly incidence, lethality and mortality rates in the periods before and after the first vaccination peak of the first dose. To develop the tables, graphs and perform statistical tests, the R Core Team (2023) software was used and the significance level was considered <5%.

All data were obtained from public databases (http://datasus.saude.gov.br/), and as this is a public domain source of data, with no possibility of identification, this study did not require approval from an ethics committee, in accordance to the Brazilian standards present in Resolution nº510/2016 of the National Health Council [18].

## Results

**Fig 1:**
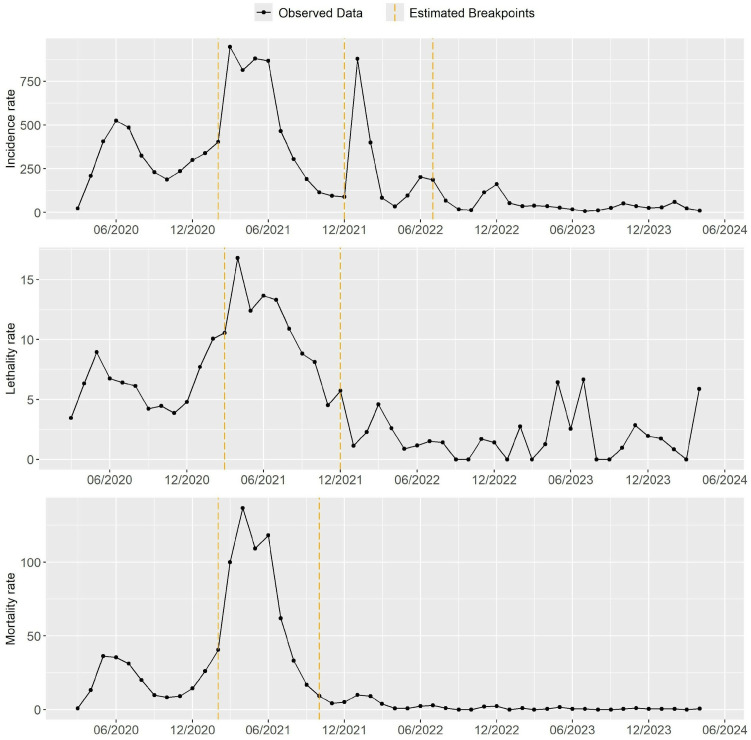
Behavior of incidence, lethality and mortality rates of pregnant and postpartum women with SARS caused by SARS-CoV-2, between the periods from March/2020 to April/2024 in Brazil.

During the study period, it was observed that the incidence of SARS-CoV-2 in pregnant and postpartum women in Brazil showed significant temporal variations, before and after the introduction of the vaccine. It was possible to identify points of change in the trend’s timeline regarding the incidence, lethality and mortality rates. For the incidence, three points of change in trend were estimated, in February 2021, December 2021 and July 2022. For the lethality, the structural change points in the series occur in March and December 2021. And for the mortality rate, the estimates indicate a change in trend in February and October 2021 (Fig 1)

**Fig 2:**
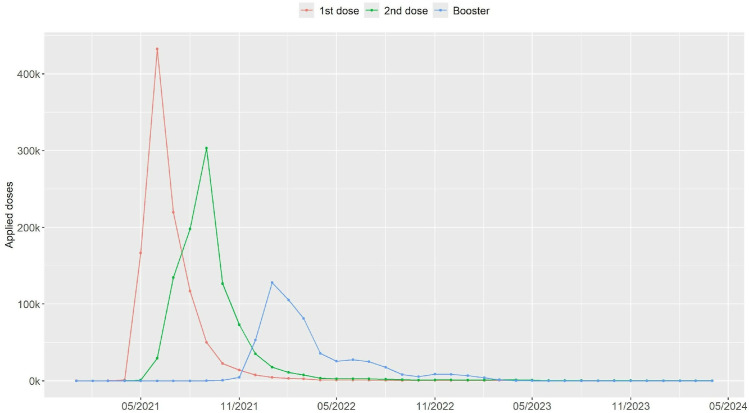
Doses of the Covid-19 vaccine administered to pregnant and postpartum women, from January/21 to April/24 in Brazil.

As for the vaccination against Covid-19 in pregnant and postpartum women, the data analyzed showed that during the study period, 2,569,702 doses of the Covid-19 vaccine were administered, considering the 1st and 2nd doses, and the booster dose. Vaccination of the first dose increased rapidly in May 2021, with the highest monthly peak of application in June 2021. Vaccination of the second dose had the highest number of applications in September 2021 (Fig 2).

The booster dose showed similar behavior, with the number of doses applied increasing rapidly from the time they were made available, reaching a peak in January 2022 and gradually decreasing in the following months. It can be seen from the graphs that the peaks of application of the second dose and booster dose did not reach the same magnitude of applications as the first dose (Fig 2).

When comparing the rates analyzed between the pre and post-vaccination periods, it can be seen that in the pre-vaccination period, no rate showed a significant trend, possibly due to the instability of the period itself, involving many fluctuations. However, in the post-vaccination period, the three rates (incidence, lethality and mortality) presented a value of <0, indicating a decreasing trend with a significant p-value (Table 1).

**Table 1:** Trends of the temporal series of Incidence, Lethality and Mortality rates due to Covid-19 in pregnant and postpartum women in the pre- and post-vaccination periods identified by the Mann-Kendall test; Chow test for structural change.

Indicators	Vaccination Period	Mann-Kendall $\begin{array}{c} \left(\tau \right) \end{array}$	P-value (Mann-Kendall)	Chow-Test	P-value (Chow)
Incidence	Pre-vaccination	0.16	0.4551	6.39	0.0036
	Post-vaccination	−0.63	<0.001		
Lethality	Pre-vaccination	0.06	0.8034	9.96	0.0003
	Post-vaccination	−0.49	<0.001		
Mortality	Pre-vaccination	0.06	0.8034	7.60	0.0014
	Post-vaccination	−0.67	<0.001		

**Fig 3:**
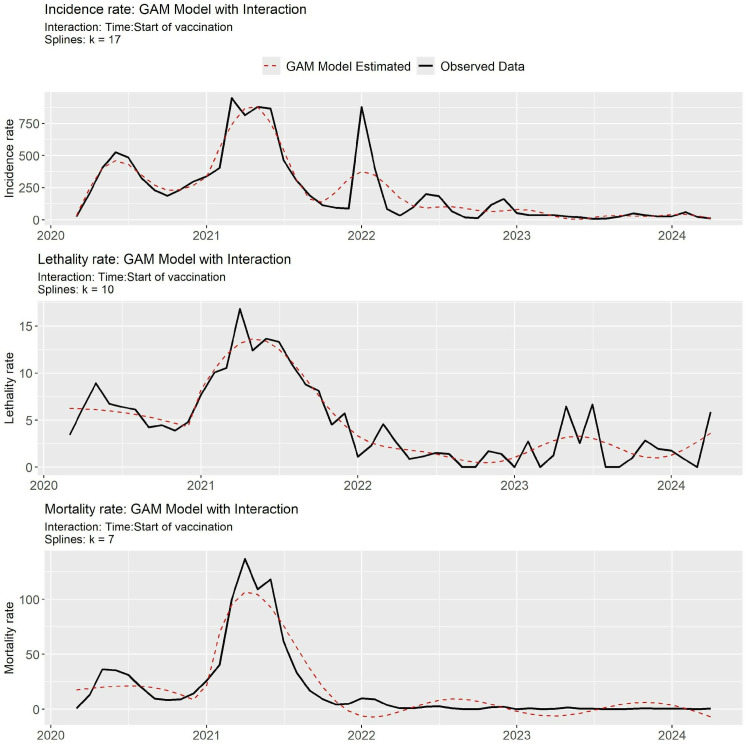
GAM models with interaction adjusted for Incidence, Lethality and Mortality rates due to Covid-19 in pregnant and postpartum women.

Fig 3 shows the adjustment of the GAM models for the three rates analyzed, indicating their respective numbers of knots (k), selected in order to maintain a reasonable similarity with the fluctuations of the series, in addition to achieving R2 values and explained deviance of around 80%. Table 2 shows the parametric coefficients estimated by the GAM models with interaction for the three rates analyzed.

**Table 2:** Estimated parametric coefficients of GAM Models with interaction for Incidence, Lethality and Mortality rates due to Covid-19 in pregnant and postpartum women.

Indicators	Coefficients	Estimate	Std. Error	t value	Pr(>|t|)
Incidence	Intercept	−2484.93	3601.09	−0.69	0.4949
Lethality	Intercept	−3.26	13.63	−0.24	0.8119
Mortality	Intercept	−92.27	206.41	−0.45	0.6573

For both lethality and mortality rates, time alone did not show a significant effect on reducing the rates, which only occurs when there is an interaction effect between time and the start of vaccination. For the incidence rate, the passage of time is significant for the change in behavior of the series, but the interaction with the start of vaccination was not significant (Table 3).

**Table 3:** Approximate smoothing terms of GAM Models with interaction for Incidence, Lethality and Mortality rates due to Covid-19 in pregnant and postpartum women.

Indicators	Coefficients	EDF	Ref.df	F value	Pr(>|F|)
Incidence	s(Time)	13.00	14.56	3.01	0.0036
	s(Time):Post-vaccination	2.15	2.17	0.26	0.7956
Lethality	s(Time)	1.59	1.82	0.36	0.7556
	s(Time):Post-vaccination	7.85	8.35	4.54	<0.001
Mortality	s(Time)	1.54	1.79	0.24	0.8307
	s(Time):Post-vaccination	6.93	6.97	12.47	<0.001

**Fig 4:**
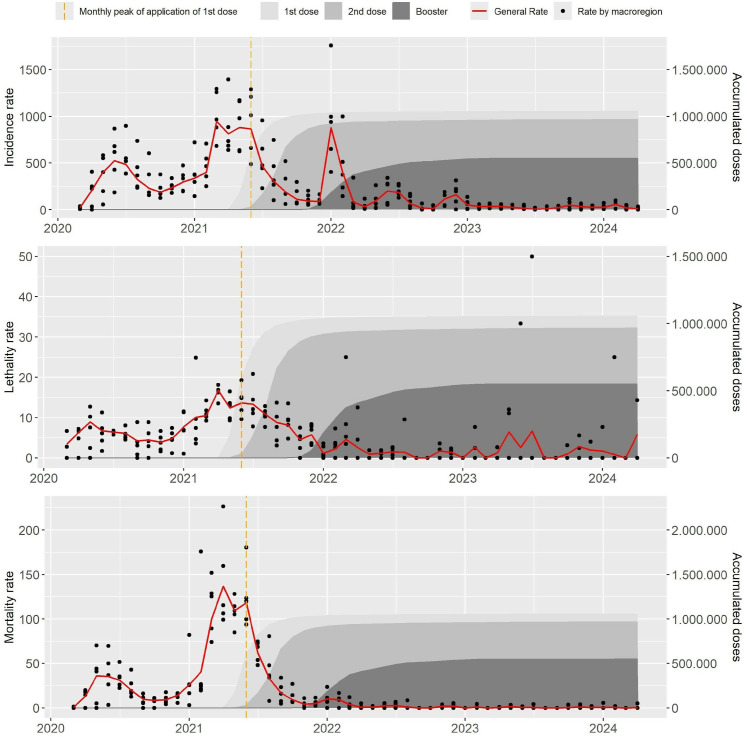
Accumulated doses of vaccine against Covid-19 and Incidence, Lethality and Mortality rates in pregnant and postpartum women by brazilian macroregion.

Fig 4 shows the curves of accumulated vaccine doses, together with the behavior of incidence, lethality and mortality in each Brazilian macro-region. The five points in each month/year of the graph correspond to the rates in the North, Northeast, South, Southeast and Central-West regions of Brazil, where it is possible to observe great variability in the rates.

The dashed line indicates the first monthly peak in the application of the first dose of the Covid-19 vaccine, and it is possible to see that the decline in incidence, lethality and mortality rates begins to occur concomitantly when the application of the 1st dose of the Covid-19 vaccine begins to strengthen (Fig 4).

A moderate to strong negative correlation was found between the number of accumulated doses of the first dose of the vaccine and the lethality and mortality rates. This result indicates that the accumulation of doses of the COVID-19 vaccine is related to the decrease in the rates analyzed, in which the accumulation of doses is capable of explaining 25.87% to 52.48% of the observed variation, depending on the rate and dosage of the vaccine. The accumulated doses of the COVID-19 vaccine were capable of explaining 39.18% of the variation observed in the incidence rate, 43.34% of the lethality rate and 34.81% of the mortality rate (Table 4).

**Table 4:** Pearson correlation coefficients between accumulated Covid vaccine doses and Incidence, Lethality and Mortality rates in brazilian pregnant and postpartum women.

Indicators	Accumulated Doses	Rho $\begin{array}{c} \left(\rho \right) \end{array}$	P-value	$\begin{array}{c} \rho^{2} \end{array}$
Incidence	1st dose	−0.55	<0.001	30.07%
	2nd dose	−0.64	<0.001	40.66%
	Booster	−0.64	<0.001	40.84%
	All doses	−0.63	<0.001	39.18%
Lethality	1st dose	−0.53	<0.001	28.42%
	2nd dose	−0.69	<0.001	47.14%
	Booster	−0.72	<0.001	52.48%
	All doses	−0.66	<0.001	43.34%
Mortality	1st dose	−0.51	<0.001	25.87%
	2nd dose	−0.63	<0.001	39.78%
	Booster	−0.57	<0.001	32.03%
	All doses	−0.59	<0.001	34.81%

Considering the monthly peak of application of the first dose of the Covid-19 vaccine in pregnant and postpartum women as the cut-off point, the results indicate that the average monthly incidence rates of cases decreased from 420 to 139 pregnant women per 100,000 live births. The fatality rate decreased from 7.53 to 3.36 per 100 cases and the mortality rate decreased from 39.42 to 8.34 per 100,000 live births. The ANOVA showed statistical significance for all three indicators, and the Tukey test corroborated the results, showing a difference between the monthly averages before and after the first vaccination peak of the 1st dose (Table 5).

**Table 5:** ANOVA and Tukey’s post-hoc to compare the average monthly rates of Incidence, Lethality and Mortality before and after the first monthly peak of application of the Covid-19 vaccine in brazilian pregnant and postpartum women.

Indicators	First monthly peak of vaccination period	Average Monthly Rate	ANOVA	P-value	Tukey
Incidence	Before	420.73	15.64	0.00025	a
	After	139.14			b
Lethality	Before	7.53	13.00	0.00074	a
	After	3.36			b
Mortality	Before	39.42	11.76	0.00125	a
	After	8.34			b

## Discussion

Given the importance of monitoring changes in the impact of Covid-19 and the effect of vaccination on pregnant and postpartum women, this study analyzed the incidence of Severe Acute Respiratory Syndrome, lethality and mortality of Brazilian pregnant and postpartum women infected with SARS-CoV-2, before and after vaccination against Covid-19. Our findings indicate that vaccination against Covid-19 influenced the reduction in the incidence of SARS, lethality and mortality in pregnant and postpartum women infected with SARS-CoV-2 in Brazil.

Between March 2020 and April 2024, which corresponds to the study time period, there were three epidemic waves of Covid-19 among pregnant and postpartum women. The 2021 epidemic wave, which corresponds to the second wave of the Covid-19 pandemic, was the one with the highest rates of severe Covid-19 among the obstetric population in Brazil, and in other countries [19,20].

One of the possible causes investigated for this increase in severe cases of Covid-19 among the obstetric population was the emergence of new variants of the SARS-CoV-19. It is known that in Brazil, during the second wave of the pandemic, there was a predominance of the Gamma variant, which presented higher rates of virulence, transmissibility and mortality, with the potential to lead to more severe cases of Covid-19 in pregnant and puerperal women [21].

A nationwide study carried out with data from the SIVEP-Flu of the Brazilian Ministry of Health, showed that pregnant and postpartum women had a higher risk (OR: 2.60; 95% CI: 2.28–2.97) of death from Covid-19 in the first months of 2021, when compared to the pandemic period of 2020, and almost double the risk of death when compared to men (OR: 1.31; 95% CI: 1.30–1.32) and non-pregnant women (OR: 1.44; 95% CI: 1.42–1.46) [21]. Other countries have also seen a substantial increase in hospitalizations of pregnant and postpartum women with severe Covid-19 [22].

In the second half of January 2021, vaccination against Covid-19 began in Brazil, however, it was only during the sixth edition of the vaccination operational plan, published on April 28, 2021, that pregnant and puerperal women were included among the priority groups [23]. Soon after, in mid-May, vaccination was suspended for pregnant and postpartum women without comorbidities, which was resumed only in early July 2021 [24,25]. This scenario shaped the first months of the Brazilian Covid-19 epidemic in 2021.

After the vaccine was made available to pregnant and postpartum women, the incidence of SARS-CoV-2, mortality and lethality rates began to decline, in addition to evidence of an inverse correlation between the evaluated indicators and the accumulated doses of the vaccine. Although no studies were found that analyzed the impact of vaccination against Covid-19 on morbidity and mortality indicators among the obstetric population, there are some studies carried out with the general population, which demonstrate similar results [10–12].

An ecological study that analyzed the general effect of vaccination against Covid-19 in the Brazilian population over 18 years of age, identified that there was a weekly trend towards an increase in morbidity and mortality indicators associated with Covid-19 in the pre-vaccination period, and a reduction in the post-vaccination period. In addition to that, it showed that the hospitalization and mortality rate had a strong inverse association with vaccination coverage [10].

In the United States, a negative association was found between the vaccination, incidence rates (r² = 35.3%) and hospitalization (r^2^ = 20.8%), in which the additional increase of 1 vaccinated individual per 100 inhabitants, reduced the incidence rate by 0.7% when vaccinated with one dose of the vaccine, and 1.1% with two doses. Given this scenario, it was estimated that vaccination reduced the number of new cases by 4.4 million and 0.12 million hospitalizations [11]. Another study, also carried out in the United States, identified that a 10% improvement in vaccination coverage was associated with an 8% and 7% reduction in mortality and incidence rates, respectively [13].

Another study that investigated the effect of vaccination on Covid-19 infection rates in eight countries (Israel, United Arab Emirates, Chile, Hungary, Qatar, Serbia, USA and United Kingdom), showed that in the USA and the United Kingdom, rates of Covid-19 infection began to reduce immediately after the start of vaccination, and in the other countries studied, there was a peak in the infection rate after the start of vaccination, followed by a decrease as the vaccination rate increased [12].

Approximately eight months after the vaccine was made available to the obstetric public in Brazil, a period in which the circulation of the Ômicron variant prevailed in the country (January to April 2022), a third epidemic wave of Covid-19 was observed, with a sudden increase in the incidence of SARS among pregnant and postpartum women. This event might have happened due to the decrease in the effectiveness of vaccines against the new variants, associated with low coverage of the booster dose [26].

It was also evident that this sudden increase in the incidence of SARS was accompanied by a slight increase in hospital mortality and lethality. A study that tested the response of antibodies induced by the Pfizer and Moderna vaccines, protecting against the variants in pregnant women, identified that despite the Omicron variant being considerably more resistant to neutralizing antibodies, the ones induced by the vaccine continued to offer protection against serious forms of the disease. Thus, despite the loss of protection against transmission, vaccines continued to play a key role in mitigating the disease in pregnant women [27].

It was also observed in the present study that the vaccination of the second dose, and chiefly the booster dose, did not reach the same magnitude of applications of the first dose. Despite several studies demonstrating the safety of the vaccine during pregnancy and the postpartum period, there is still significant vaccine hesitancy among pregnant and postpartum women [28–31]. This hesitation exposes the obstetric population to the severe evolution of Covid-19 and unfavorable outcomes, as well as to obstetric complications already documented in pregnant women infected with SARS-CoV-2, such as premature birth, low birth weight and stillbirth [32].

Vaccine hesitancy among the obstetric population is significantly related to the lack of knowledge about the impacts of vaccination on pregnancy, fetal development and the well-being of the child [31,33,34]. In Brazil, the large-scale circulation of fake news about vaccines discouraged the adherence of segments of the Brazilian population to vaccination [35]. In view of this scenario, there is a need for targeted and multifaceted efforts to increase confidence in vaccines, especially for populations vulnerable to the severe evolution of the disease, such as pregnant and postpartum women.

The study has limitations, such as the use of secondary data that are subject to delay in reporting cases and deaths from Covid-19, which in turn may underestimate the analyzed indicators. However, it is emphasized that the information system used is currently the official database of the Brazilian Ministry of Health for recording SARS cases and deaths, and has been the main source of data in the country during the Covid-19 pandemic.

The findings of this study indicate that vaccination against Covid-19 in the obstetric population had a positive effect on reducing the incidence rates of SARS, lethality and mortality in pregnant and postpartum women infected with SARS-CoV-2 in Brazil. In this sense, this study contributes with findings of national and international interest, while providing support for the planning of public policies that favor vaccination against Covid-19 in the obstetric population, and finally contributing to the reduction of the incidence of SARS, maternal mortality and lethality.
